# Integrative Multi-Omics Deciphering of Gu Shu Kang Granules: A Comprehensive Systems Biology Approach to Unraveling Molecular Mechanisms in Sarcopenia-Osteoporosis Intervention

**DOI:** 10.2174/0118715303448084251113101628

**Published:** 2025-11-26

**Authors:** Xiangran Cui, Zhitong Yu, Deyu Wang, Yantong Liu, Weiren Wang, Hai Teng, Miao Yu, Shixuan Wang, Hongfei Liu, Wei Wei

**Affiliations:** 1 Department of Orthopedics, Second Affiliated Hospital of Liaoning University of Traditional Chinese Medicine, Shenyang, 110167, P. R. China

**Keywords:** Muscle-bone crosstalk, proteomic mass spectrometry, osteosarcopenia (OS), gushukang granules, traditional Chinese medicine (TCM), osteoporosis

## Abstract

**Introduction:**

Sarcopenia is a degenerative musculoskeletal disease affecting the elderly, significantly impairing patients' quality of life and challenging modern medicine. This study innovatively combines Traditional Chinese Medicine (TCM) theories with modern medical research to explore the mechanisms by which Gushukang granules address sarcopenia.

**Methods:**

The research integrated multi-dimensional research methods, including network pharmacology, metabolomics, and animal experiments, to comprehensively investigate the scientific mechanisms of Gushukang granules' intervention in sarcopenia.

**Results:**

Network pharmacology analysis identified multiple potential targets related to muscle growth and repair. UPLC-Q-TOF MS technology tracked metabolic pathways, while animal experiments verified that Gushukang granules precisely regulate *muscle* metabolic balance by modulating key signaling pathways involved in protein synthesis and degradation.

**Discussion:**

The findings demonstrate the potential of integrating traditional and modern medical approaches in addressing age-related muscle degradation, providing scientific validation for TCM treatment of sarcopenia.

**Conclusion:**

This study establishes a model for modernizing TCM research, offering solid scientific evidence for comprehensive intervention of chronic diseases in the elderly and highlighting the TCM concept of “preventing disease before its onset” in modern medical translation.

## 1 INTRODUCTION

Osteoporosis (OP) and Sarcopenia (SP) are two conditions that significantly impact the health of older adults. OP is characterized by decreased bone density and deterioration of bone microarchitecture, leading to fragile bones and a significantly increased risk of fractures [1]. SP, on the other hand, manifests as a reduction in muscle mass and strength, affecting functional ability and quality of life [2]. These two conditions often coexist, with case studies showing a gradual decline in muscle and bone health as people age. Epidemiological studies indicate that the prevalence of SP rises significantly among the elderly population, reaching as high as 29% among community dwellers and increasing to 33% in long-term care facilities. Among those over 80 years old, the prevalence can be as high as 50% [3]. At the same time, with the accelerating aging of the population, the incidence of OP is also on the rise, affecting more than 70% of individuals over 80 [4]. Over 10% of men and 15% of women are closely affected by fragility fractures and complications related to osteosarcopenia. To explain the complex relationship between the two, in 2009, Neil Binkley and colleagues first proposed the concept of “Osteosarcopenia” (OS), emphasizing the interaction between muscle and bone and their integration in pathophysiology [5]. This concept provided a new perspective to the medical community, helping researchers understand the relationship between muscle and bone holistically and promoting the development of research on the musculoskeletal system [6, 7]. With the continued increase in prevalence of this condition, the heightened fracture risk among older adults, and the accompanying high morbidity and mortality rates, osteosarcopenia poses a serious threat to patients' quality of life in later years and increases the burden on public health.

Muscles and bones both originate from mesenchymal stem cells, sharing a high degree of homology and being influenced by commonly regulated differentiation genes [8]. Since they share the same origin and are anatomically close, muscles and bones are interconnected, responding together to similar mechanical loads and the combined effect of bioactive factors. Studies show that mechanical loading can significantly improve the quality and strength of both muscles and bones [9, 10]. Muscle contractions induced by loading not only stimulate the growth of skeletal muscle but also affect bone geometry and bone density [11, 12]. Conversely, reduced loading leads to neuromuscular degeneration and bone loss. Skeletal muscle regulates bone metabolism through mechanical stimulation, providing kinetic energy and protection to bones while enhancing bone strength. Endocrine disorders, such as diabetes, thyroid dysfunction, vitamin D deficiency, sex hormone imbalances, malnutrition, and obesity, are major causes of sarcopenia and osteoporosis [10, 13, 14]. As sarcopenia progresses, the decline in muscle strength and mass accelerates the development of osteoporosis. Conversely, reduced bone strength may result in muscle atrophy, a decrease in muscle cells, and functional deterioration [15, 16]. In this process, skeletal muscle is not only responsible for movement but also serves as a vital endocrine organ, capable of regulating the metabolism of other body tissues and influencing the function of distant organs through autocrine, paracrine, and endocrine mechanisms [17-19].

In traditional Chinese medicine, symptoms related to sarcopenia-osteoporosis are generally classified under the categories of bone atrophy and bone impediment [20, 21]. Gushukang Granules, as Chinas first approved pure traditional Chinese medicine formulation for treating osteoporosis, have ushered in a new era of using Chinese herbal medicine for the prevention and treatment of osteoporosis [22]. Its herbal components include *Epimedium, Rehmannia glutinosa, Drynariae Rhizoma, Astragalus, Salvia miltiorrhiza, black fungus,* and cucumber seeds, which together function to tonify the kidneys and vital energy, promote blood circulation, and strengthen bones [23, 24]. Research has shown that Epimedium and Astragalus effectively reduce levels of inflammatory factors such as IL-6 and TNF-α in the body. In contrast, the extract of *Drynariae Rhizoma*, naringin, promotes osteoblast differentiation through pathways involving estrogen receptors [25-27]. Compared to commonly used drugs in modern medicine, Gushukang Granules offer advantages such as ease of administration, stable therapeutic effects, and minimal side effects, and have been widely applied in clinical practice. Studies also indicate that Gushukang Granules can reduce osteocyte apoptosis in *osteoporotic mice*, increase bone density and mineral content, effectively regulate bone metabolism, and improve clinical symptoms [28, 29]. Additionally, some researchers have demonstrated that Gushukang promotes osteoclast apoptosis and inhibits bone resorption in *osteoporotic rats*, thereby slowing bone loss and treating osteoporosis [22].

In the clinical treatment of osteoporosis, we observed that Gushukang granules have a certain therapeutic effect on symptoms such as muscle soreness and weakness in the lower back and knees, with patients reporting an improvement in muscle strength. Traditional Chinese Medicine (TCM) theory states that “the spleen governs the muscles” and “the kidney governs the bones” [30-32]. Considering the multi-gene pathway regulation characteristics of osteoporosis and sarcopenia, this highlights the interplay of multi-organ, multi-system, and multi-target systemic metabolic diseases [33, 34]. Although the efficacy of Gushukang granules in treating osteoporosis has been verified, current reports on their effects on muscle-related issues remain limited. Traditional Chinese medicine often involves multiple levels, dimensions, and targets. Proteomic mass spectrometry can reveal protein expression levels, thereby providing a better understanding of the mechanisms of TCM [35, 36]. When Chinese medicine is notably effective, proteomics can identify signaling proteins involved in disease processes and clarify the pathways through which the medicine acts [37, 38]. Therefore, we will employ molecular biology techniques to profoundly investigate the effects of Gushukang granules on sarcopenia and identify their common targets and pathways. This research will not only help improve treatment efficacy but also hold significant practical value for understanding the pathogenesis.

**Fig. (1):**
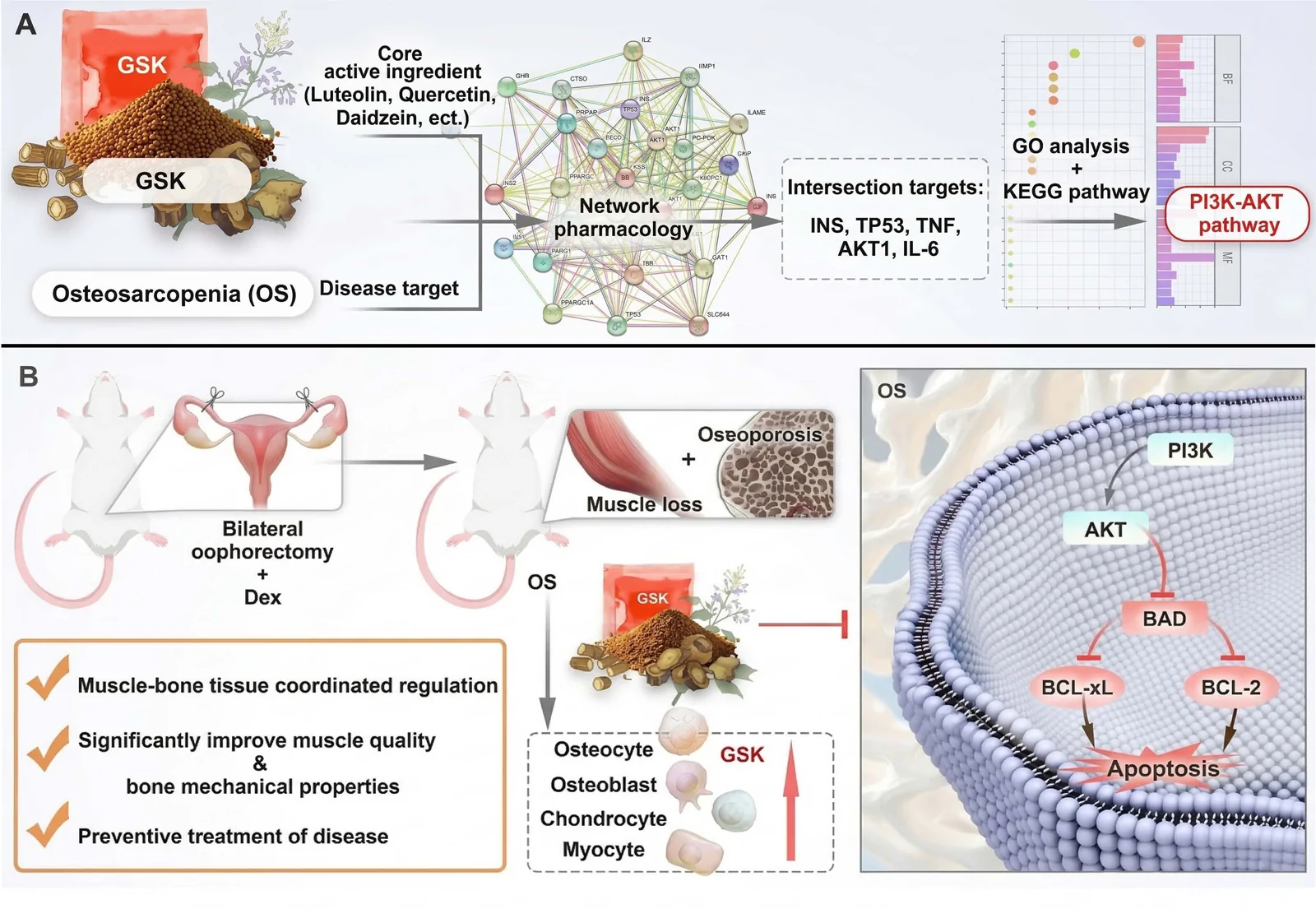
(**A**) Used network pharmacology to screen the targets related to Gushukang granules and the disease, as well as the pathways of related biological functions. (**B**) Obtained sarcopenia-*osteoporosis rats;* Gushukang granules successfully promoted osteogenesis and myogenesis *via* PI3K-Akt.

To better explore the effects of Gushukang on sarco-osteoporosis, this study employed network pharmacology and molecular docking methods to screen targets and found that the key targets exhibited strong binding affinities with active compounds. These factors may be critical for treating sarco-osteoporosis. However, due to the complexity of sarco-osteoporosis and the limitations of network pharmacology and molecular docking techniques, we also conducted mass spectrometry analysis of Gushukang extracts and animal experiments to verify its mechanism of action and provide a basis for clinical treatment (Fig. **1**).

## 2 MATERIALS AND METHODS

### 2.1 Construction of a Database of the Components of Gushukang Granules (GSK)

Gushukang Granules (GSK) include five active ingredients, including Danshen (SM), Huangqi (AM), Duzhong (DF), Epimedium (EP), and an additional ingredient. The compound data for SM, AM, DF, EP, and GC were sourced from the Traditional Chinese Medicine Systems Pharmacology Database (TCMSP) (http://lsp.nwu.edu.cn/tcmsp.php). After removing duplicate data, a total of 81 compounds were identified in the GSK dataset.

### 2.2 Screening of the Active Ingredients in GSK

Drug likeness is a qualitative measure utilized in drug development to evaluate how potential drug candidates align with and enhance pharmacokinetic properties and characteristics. Oral bioavailability serves as an indicator of a molecule's suitability as a therapeutic agent, representing the proportion of an orally administered drug that successfully enters the bloodstream, which is reflected in the integration of ADME processes. To pinpoint the active ingredients from GSK, components were selected for further analysis based on published literature and data obtained from the TCMSP database, specifically those that met the criteria of having an Oral Bioavailability (OB) of 30% or higher and a Drug-Likeness (DL) score of 0.18 or above.

### 2.3 Related Targets of Osteoarthritis (OA) and Sarcopenia (SP)

Data on target genes related to OA and SP were compiled from several sources. DisGeNET (https://disgenet.com/) is a comprehensive online platform that offers detailed biochemical and pharmacological insights into drugs, including their mechanisms of action and specific targets. GeneCards (http://www.genecards.org/) is a unified database that includes both annotated and predicted information on genes, which was searched using the terms “osteoarthritis” and “sarcopenia”. The Online Mendelian Inheritance in Man^®^ (OMIM) database (http://www.omim.org/) serves as a thorough research tool for exploring human genes and genetic phenotypes. After consolidating the information from these three databases and eliminating duplicate targets, a total of 4,621 targets were identified as being associated with OA, while 100 targets were linked to SP.

### 2.4 Construction of the Pharmacological Networks

To improve the reproducibility of the compound-target mapping workflow, we followed four steps to construct the network. Firstly, a compound-target network was established by connecting compounds with predicted targets having a degree greater than three. Secondly, compound targets were connected to other human proteins to construct a compound-target protein-protein interaction (PPI) network. Thirdly, known OA- and SP-related targets were connected with predicted other human protein targets to construct an OA and SP target PPI network. Finally, a PPI network including GSK, OA, and SP targets was constructed by intersecting the compound PPI network with the OA target PPI network. Network analysis and visualization were performed using Cytoscape version 3.7.0 (http://www.cytoscape.org/), ensuring efficient graphical and schematic representation, thereby enhancing the transparency and reproducibility of the study.

### 2.5 Gene Ontology (GO) and Pathway Enrichment Analysis

We employed the Gene Ontology (GO) database (http://geneontology.org/), which encompasses terms related to biological processes, cellular components, and molecular functions, to explore potential biological mechanisms through high-throughput genomic or transcriptomic data. Additionally, we utilized the Kyoto Encyclopedia of Genes and Genomes (KEGG) database (https://www.kegg.jp/), a resource that helps in identifying the systematic functions and biological significance of various candidate targets. For the functional annotation of GO terms and the analysis of KEGG pathways, we utilized the clusterProfiler R package from Bioconductor, designed specifically for gene cluster enrichment analysis.

### 2.6 Molecular Docking Methods

To enhance the reproducibility of the compound-target mapping workflow, we employed AutoDock Vina as the molecular docking software. The specific parameters were as follows: We used an empirical free energy scoring function to calculate the binding affinity between compounds and the target; the exhaustiveness was set to 20 to ensure a comprehensive search for possible binding conformations; the grid box size was 20 Å x 20 Å x 20 Å, with the center positioned at the active site of the target. These settings ensured the reliability of the docking results, and the standardized procedure contributes to the transparency and reproducibility of the research.

### 2.7 UPLC Method

UPLC-MS/MS analyses were conducted using a Waters Xevo TQ-S mass spectrometer coupled with a Waters ACQUITY UPLC system. The chromatographic separation was achieved using a C18 column, with a mobile phase consisting of water and acetonitrile, both containing 0.1% formic acid. The analysis utilized gradient elution, and the mass spectrometer operated in both positive and negative ionization modes to effectively identify and quantify the active compounds in the samples.

### 2.8 Animals

Animal experiments were conducted in accordance with the NIH Guide for the Care and Use of Laboratory Animals and were approved by the Institutional Review Board of Liaoning University of Traditional Chinese Medicine (IRB No. LZYY240406). Sixty 6-week-old female *SPF-grade SD rats* (160 ± 16 grams) were purchased from Liaoning Changsheng Biotechnology Co., Ltd., with the experimental animal production license number: SCXK (Liao) 2022-0001. After acquisition, the animals were housed in the SPF-grade animal facility of the Experimental Animal Center at Liaoning University of Traditional Chinese Medicine, with a license number: SYXK (Liao) 2022-0009. The *rats* were fed a standard pellet diet with free access to food and water, under a 12-hour light-dark cycle.

#### 2.8.1 Animal Experiment Grouping and Model Establishment

*SD rats* were randomly divided into four groups: the control group (Control), the Osteoporosis (OS) model group (OS), the OS + Bone Health Group (OS+GSK), and the OS + Estradiol Group (OS+E2). The modeling method involved bilateral ovariectomy combined with dexamethasone sodium phosphate injection. After surgery, *rats* were given intramuscular injections of penicillin sodium (80,000 units per *rat)* daily for 3 consecutive days to prevent infection. One week later, *rats* in the OS model group (OS), OS + Bone Health group (OS+GSK), and OS + Estradiol group (OS+E2) received intraperitoneal injections of dexamethasone sodium phosphate injection (DXM) at a dose of 1 mg/(kg/day) for 2 consecutive weeks. After successful modeling, drug intervention commenced. The OS+GSK group was gavaged daily with 2.8 g/kg of Bone Health granule aqueous solution, and the OS+E2 group was gavaged daily with 0.104 mg/kg estradiol valerate tablets. The other groups received 10 mL/kg of saline by gavage daily. The treatments continued for 3 months. Based on body surface area conversion, the equivalent human-rat dose of the Bone Health granule aqueous solution administered to *rats* was calculated as 2.8 g/kg.

#### 2.8.2 SD Rat *Bone and Muscle Sample Collection*

After 3 months of drug intervention, the *rats* were anesthetized, and specimens were collected. Once anesthesia took effect, the blood was drawn from the abdominal aorta, and the serum was separated and stored in an ultra-low temperature freezer. The right femur, right gastrocnemius muscle, and right soleus muscle were quickly dissected, and the surrounding soft tissues were carefully removed. The tissues were rinsed with pre-cooled PBS, placed into cryovials, and stored in an ultra-low temperature freezer. The left femur, left gastrocnemius muscle, and left soleus muscle were also wholly and quickly dissected, with surrounding soft tissues carefully removed. They were then rinsed with pre-cooled PBS, and the surface moisture was absorbed using clean filter paper. The wet weight of the left gastrocnemius and soleus muscles was measured using a balance, after which the tissues were placed into 10 mL centrifuge tubes and fixed with paraformaldehyde. The uterus, liver, and kidneys were rapidly dissected, with surrounding soft tissues carefully removed. After rinsing the organs with pre-cooled PBS, surface moisture was blotted using clean filter paper. The wet weight of each organ was recorded, and the tissues were transferred to cryovials for storage in an ultra-low temperature freezer.

#### 2.8.3 *Histological Observation of Muscle in* SD Rats

The samples were fixed in a 10% formaldehyde solution for a duration of 48 hours. Following fixation, they were rinsed with both distilled and deionized water, then subjected to decalcification using ethylenediaminetetraacetic acid (EDTA). Once decalcified, the samples were embedded in paraffin, and sections measuring 5 μm in thickness were cut from the center of each sample using a microtome. Subsequently, the sections were stained with Hematoxylin and Eosin (H&E). Optical micrographs of the prepared slices were captured using a microscope.

#### 2.8.4 Immunohistochemical Staining

The bone tissue sections, which had been deparaffinized, were washed three times with Phosphate-Buffered Saline (PBS) before undergoing pretreatment with 0.1% trypsin for 15 minutes to improve immunoreactivity. After the washing, the samples were allowed to soak in a 0.3% hydrogen peroxide solution at room temperature for 30 minutes. Subsequently, the sections were incubated in PBS supplemented with 3% normal horse serum (diluted 1:200) at room temperature for one hour to prevent nonspecific binding. The primary antibody used was M86, applied at a dilution of 1:200 (50 μl), while biotinylated horse anti-mouse antibody served as the secondary antibody. Following another PBS wash, the sections were developed in DAB solution. Positive results were indicated by the presence of brown deposits on the surface of the cells.

### 2.9 qRT-PCR Detection of Osteogenic and Myogenic Gene Expression

RT-PCR technology was used to detect the expression of osteogenic and myogenic-related genes. Total RNA from different samples was extracted using a total RNA extraction kit (C06-01029, Bioss, CN), and 1 μg of total RNA from each sample was used for cDNA synthesis. cDNA synthesis was performed using the SureScript First-Strand cDNA Synthesis Kit (QP057, GeneCopoeia, US). BlazeTaq SYBR Green qPCR Mix (QP033, GeneCopoeia, US) was used as the qPCR master mix for the following qPCR detection. GAPDH was selected as the internal reference gene, and the osteogenic genes OGN, OCN, and IGF-1, as well as the myogenic genes IGF-1, BDNF, and IL-6, were assessed for expression levels in the gastrocnemius muscle. All gene expression results were calculated using the delta-delta Ct method(2^−ΔΔCt^).

### 2.10 Western Blotting

Gastrocnemius muscles and femurs were collected from different animal models of *rats,* and cellular proteins were extracted. Subsequently, a 12.5% separating gel and a 4% stacking gel were prepared, and the SDS/polyacrylamide gels were placed in the electrophoresis tank for sample loading, electrophoresis, and protein transfer. Subsequently, the polyvinylidene fluoride (PVDF) membranes were washed three times with Tris-Buffered Saline (TBS)-Tween 20 (TBST), each for 10 minutes. The membranes were blocked with TBST containing 5% non-fat dry milk for 2 hours. Next, the membranes were incubated with primary antibodies, including GADPH (1:5000, Ag0766, Proteintech), OGN (1:1000, 10323-2-AP, Proteintech), IGF-1 (1:500, 10375-2-AP, Proteintech), Akt (1:1000, 13039-1-AP, Proteintech), Bad (1:500, 12149-1-AP, Proteintech), bcl-2 (1:1000, 10326-1-AP, Proteintech), followed by HRP-conjugated secondary antibodies, including HRP-labeled goat anti-mouse IgG (1:5000, A9917, Sigma Aldrich) and HRP-labeled goat anti-rabbit IgG (1:5000, A0545, Sigma Aldrich). Finally, proteins were visualized using enhanced chemiluminescence reagents (Perkin Elmer) and autoradiography (ChemiDoc MP Imaging Systems, Bio-Rad). The displayed immunoblot images represent at least three independent experiments.

### 2.11 Tunel Staining

The tissue samples were fixed with 4% paraformaldehyde for 15-30 minutes, then permeabilized with 0.1% Triton X-100. After washing with PBS, the samples were incubated with the TUNEL reaction mixture at 37°C for 60 minutes in the dark. Following this, the samples were rewashed with PBS, counterstained with DAPI to visualize the nuclei, and then analyzed under a fluorescence microscope, where apoptotic cells exhibited green fluorescence.

### 2.12 Statistical Analysis

All quantitative data are expressed as mean value ± Standard Deviation (SD). Both migration and invasion assays were analyzed with one-way analysis of variance (ANOVA) followed by Tukeys post-hoc multiple comparison test. All significance tests and data plotting were completed using GraphPad Prism v9.1.0. P-values were set at four different significance levels, such as *p ≤ 0.05, **p ≤ 0.01, ***p ≤ 0.001, ****p ≤ 0.0001.

## 3 RESULTS

### 3.1 Network Pharmacology Analysis of GSK's Mechanism in Treating Osteosarcoma

**Fig. (2):**
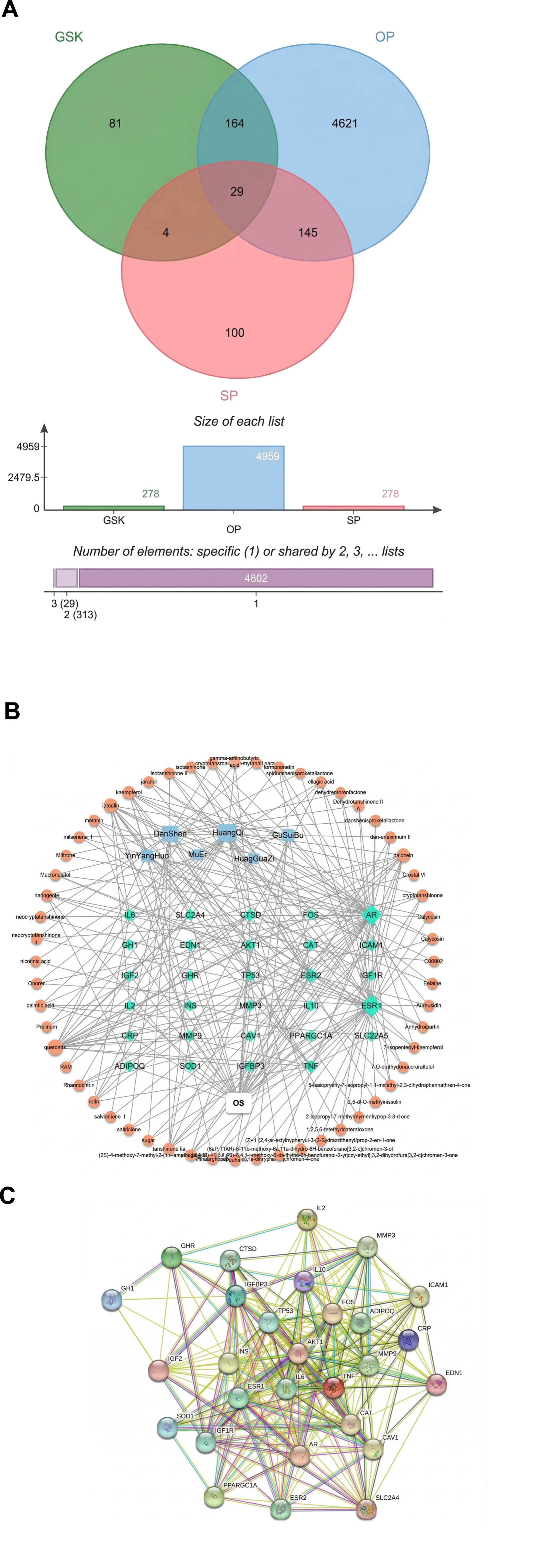
(**A**) Potential therapeutic targets of Gushukang Granules for sarcopenic osteoporosis screened through network pharmacology. (**B**) Compound compound network targets based on reverse screening of the potential targets, the putative active components of Gushukang were obtained. The “single-herb–active component–target” network was constructed using Cytoscape 3.9.0. (**C**) Cytoscape 3.9.0 analysis of the PPI network indicated that INS, TP53, TNF, AKT1, and IL6 may be important targets of Gushukang Granules in treating OS.

Using the TCMSP database, Oral Bioavailability (OB) ≥30% and Drug-Likeness (DL) ≥0.18 were selected as screening criteria to obtain active components that meet the OB and DL standards and exhibit good pharmacological activity. Through the OMIM, DisGeNET, and GeneCards databases, 4,959 OP-related targets and 278 SP-related targets were obtained; their intersection yielded 174 common targets. The intersection of the effective component targets of Gushukang granules with the OS targets resulted in 29 potential action targets (Fig. **2A**).

### 3.2 Compound-compound Network Targets

Based on reverse screening of potential action targets, the potential active components of Gushukang were obtained. The “Single Herb-Active Component-Action Target” network was constructed using Cytoscape 3.9.0 software (Fig. **2B**). Topological analysis was conducted according to degree values, identifying five key active components, such as quercetin, luteolin, daidzein, kaempferol, and tanshinone IIA. The potential action targets were uploaded to the STRING database to obtain the target interaction network. The network lines are densely interconnected and complex, indicating strong associations among the various targets. This suggests that the active components of Gushukang granules act through closely linked targets during the treatment of Osteoporosis (OS). Thicker lines represent stronger associations between targets. Cytoscape 3.9.0 software was used to analyze the PPI network to extract relevant information.

In our study of GSKs mechanism of action against osteoporosis, we used a multidimensional screening approach that integrated network topology and biological function, ultimately identifying INS, TP53, TNF, AKT1, and IL6 as potential core targets. This screening was based on degree centrality principles in CytoNCA network analysis, focusing on these targets connectivity and hub roles within the protein protein interaction network and their key regulatory functions in biological processes. These targets not only exhibit significant centrality in the network topology but also play central roles in critical physiological processes such as metabolic regulation, cell cycle, apoptosis, inflammatory response, and cell survival, providing a systematic and scientific basis for Gushukang Granules intervention in osteoporosis (Fig. **2C**).

### 3.3 GO and KEGG Enrichment Analysis of Core Targets of Gushukang Granules in the Treatment of OS

**Fig. (3):**
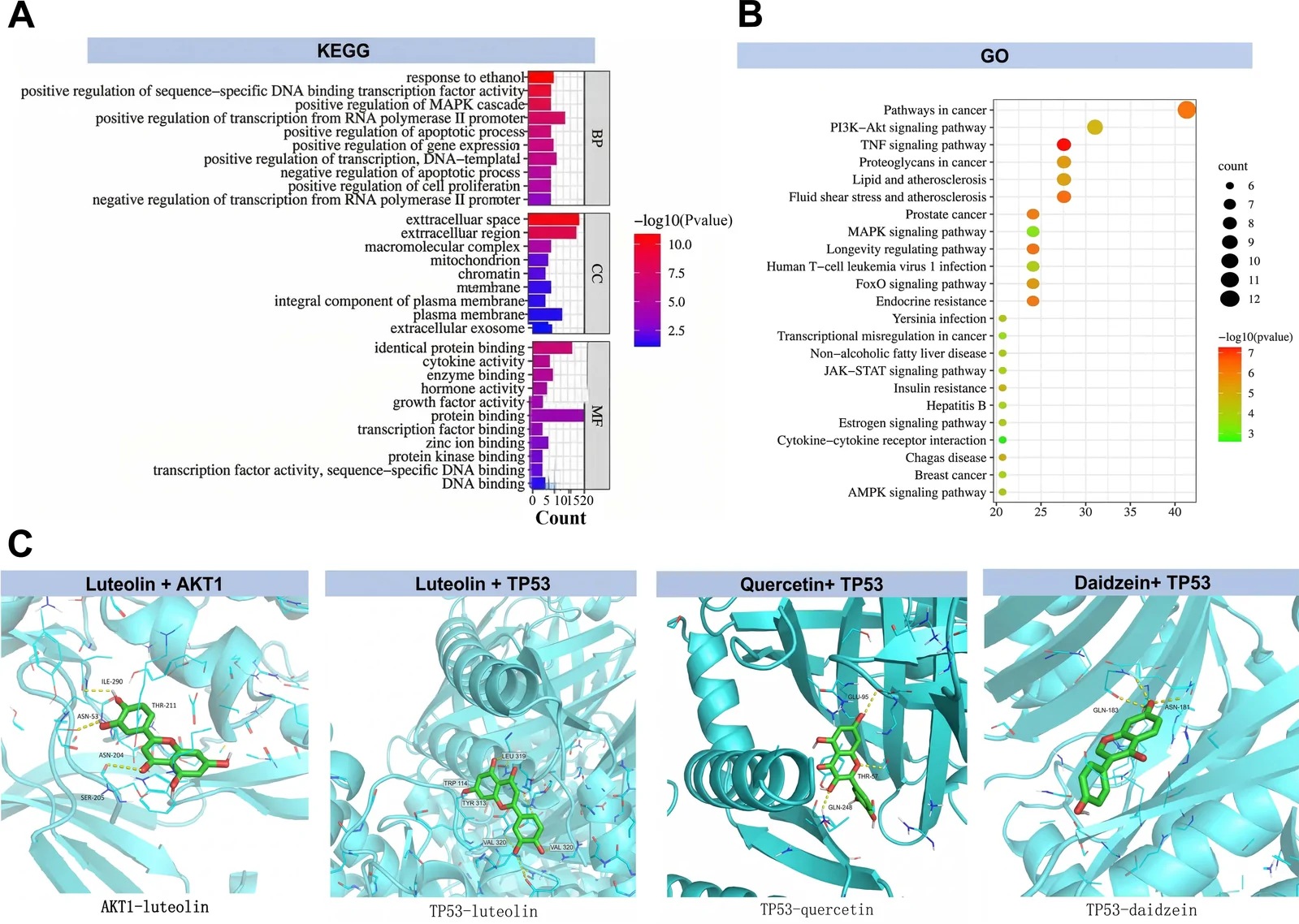
(**A**) KEGG jointly identified 83 pathways, and the top 20 were selected to draw a bubble plot, mainly involving the PI3K-Akt, Apoptosis, JAK-STAT, MAPK, Insulin, and TNF signaling pathways. (**B**) GO enrichment based on 29 potential therapeutic targets yielded 24 cellular components, 37 molecular functions, and 238 biological processes, and the top 10 items from each category were selected for visualization. (**C**) Molecular docking between core target proteins (INS, TP53, TNF, AKT1, IL6) and quercetin, flavone, and daidzein showed good binding affinity between key targets and active compounds.

Through KEGG pathway analysis, a total of 83 KEGG entries were obtained, and the top 20 pathways were selected to create a bubble chart (Fig. **3A**). The results indicated that the biological pathways through which Gushukang treats sarcopenia-osteoporosis primarily involve PI3K-Akt, Apoptosis, JAK-STAT, MAPK, Insulin, and TNF signaling pathways. The results of the GO enrichment analysis revealed that the 29 potential therapeutic targets of Gushukang granules for treating sarcopenia-osteoporosis were associated with 24 cellular components, 37 molecular functions, and 238 biological processes. The top 10 entries from each category were selected to generate analysis charts (Fig. **3B**).

### 3.4 Molecular Docking

Molecular docking was performed between core target proteins (INS, TP53, TNF, AKT1, IL6) and the active compounds quercetin, luteolin, and daidzein. The results showed that the lowest binding energies ranged from -10.1 to -5.7 kcal/mol, all ≤ -5.0 kcal/mol, indicating good binding affinity between the key targets and active compounds (Fig. **3C**). Among them, the binding energies of luteolin with AKT1 and TP53, quercetin with TP53, and daidzein with TP53 were -10.1, -8.6, -8.5, and -8.3 kcal/mol, respectively, demonstrating the best binding affinities. Optimization outputs were generated using PyMOL software (Table **1**).

### 3.5 UPLC-MS/MS Method for Chemical Profiling of GSK Particles

We employed an ultra-performance liquid chromatography-tandem mass spectrometry (UPLC-MS/MS) method to conduct chemical profiling of GSK granule samples. From the compound samples, 462 active compounds were identified, encompassing structural types such as diterpenoid saponins, triterpenoid saponins, flavonoids, flavonoid glycosides, nucleotides, aporphines, amino acids, organic acids, and others. Using Cytoscape, we constructed the GSK regulatory network and ultimately screened the main active components based on degree values, including glycyrrhetinic acid, linoleic acid, commiphora oil acid, ursolic acid, corosolic acid, betulinic acid, ricinoleic acid, jasmonic acid, asiatic acid, and ivy saponins. Most of the compounds belong to pentacyclic triterpenoids (Table **2**).

### 3.6 The Effect of GSK on Animal Body Weight and Gastrocnemius Muscle Wet Weight

**Fig. (4):**
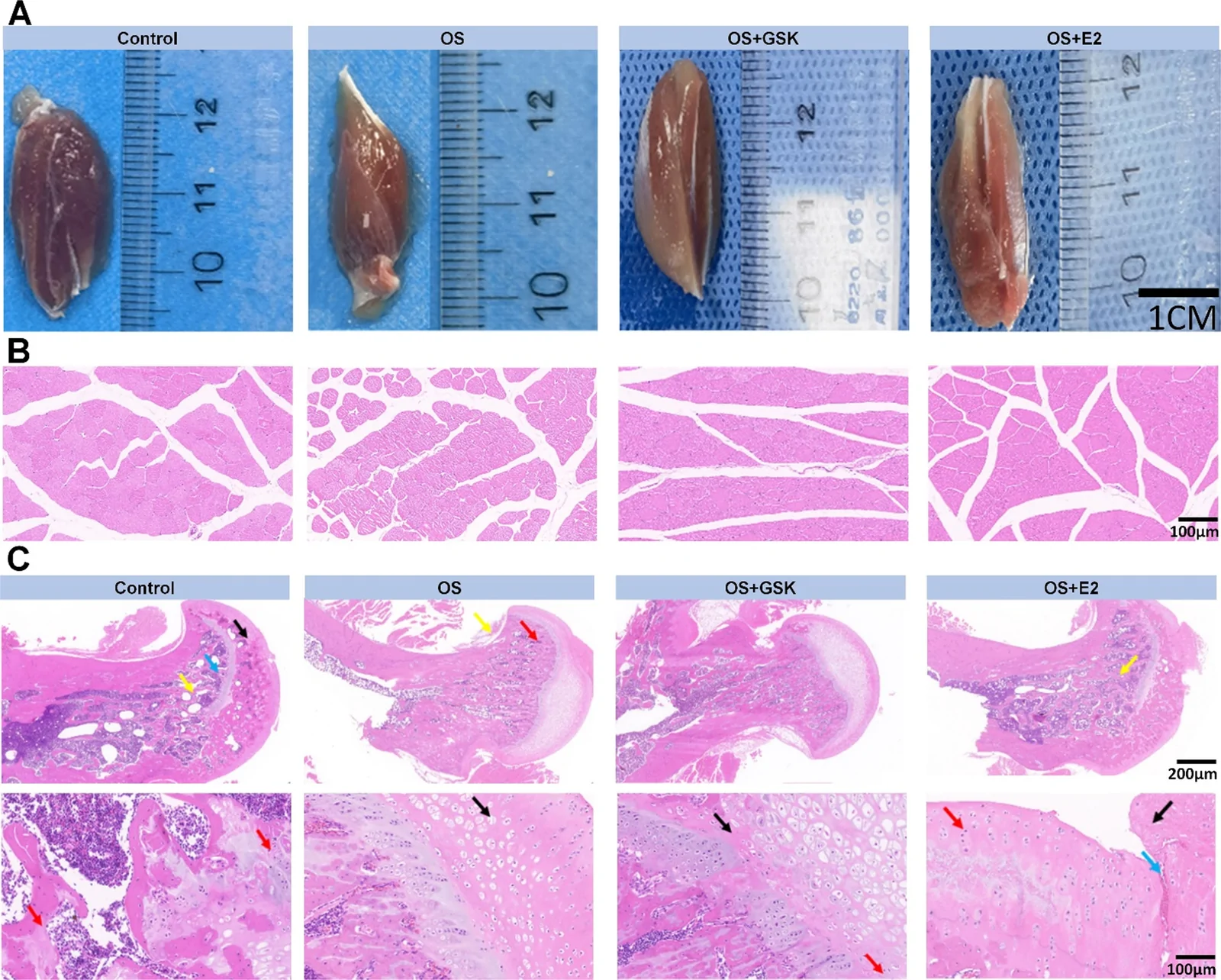
(**A**) Gross observation photos of muscles from the control group, OS group, OS+GSK group, and OS+E2 group. After the intervention, both the Gushukang granule group and the estradiol group of *rats* showed significantly increased body weight and gastrocnemius muscle mass compared with the model group (*P* < 0.05), indicating that both interventions effectively ameliorated muscle atrophy and weight loss caused by osteoporosis. (**B**) HE staining of muscle tissue. In the model group, muscle tissue exhibited pronounced pathological alterations, with marked variability in fiber size, irregular fiber morphology, and widened intercellular spaces. Myofibers appeared more rounded, with a loss of normal fascicular symmetry. In the estradiol (E2) group, muscle cells displayed regular shapes and orderly alignment, closely resembling the control group, with no morphological changes indicative of muscle loss. The Gushukang (GSK) group likewise showed well-preserved myofiber morphology, orderly arrangement, and no histological signs of muscle loss. (**C**) HE staining of skeletal tissue. In the model group, the articular cartilage surface appeared rough, with focal chondrocyte detachment leading to fissure formation. Localized defects were observed in the growth plate, and a subset of chondrocytes and osteocytes exhibited necrosis and lysis. By contrast, the femoral articular cartilage surface in the control group was smooth, with no apparent abnormalities in chondrocytes; the trabecular bone and growth plate structures were intact, and no marked inflammatory cell infiltration was noted. In the GSK group, occasional chondrocyte necrosis and lysis were observed within the growth plate, but the overall architecture of the articular cartilage and trabecular bone remained preserved, with no evident signs of inflammation.

Compared with the control group, the body weight and gastrocnemius muscle mass of *rats* in the model group were significantly decreased (*P* < 0.05), reflecting the typical pathological features of the osteoporosis model, indicating that the disease model was successfully established and resulted in significant muscle atrophy and weight loss (Table **3**). After the intervention, the body weight and gastrocnemius muscle mass of *rats* in both the Bone-Support Granules group and the estradiol group were significantly increased compared to the model group (*P* < 0.05), demonstrating that both interventions effectively improved muscle atrophy and weight loss caused by osteoporosis. The therapeutic effect of the Bone-Support Granules group was comparable to that of the estradiol group, showing good potential for treatment (Fig. **4A**).

### 3.7 Histological Staining Observation of GSK's Effects on Bone and Muscle

#### 3.7.1 Muscle and Skeletal Tissue HE Staining

In the control group, muscle tissue exhibited typical cross-sectional characteristics, with clearly observable densely arranged muscle cells, intact and uniform muscle fiber structures, and an overall normal morphology without obvious abnormalities. This indicates that the muscle tissue in the control group was healthy and functioned well, maintaining the muscle structure at a normal level. In the model group, the muscle tissue showed significant pathological changes, with muscle cells varying in size and displaying irregular shapes, along with widened intercellular spaces, indicating apparent muscle loss. The muscle cells became more rounded and lacked the normal fiber symmetry, reflecting muscle tissue degeneration and damage associated with osteoporosis-induced muscle atrophy. In the estradiol group, the muscle cells appeared regular in shape and orderly arranged, similar to the control group, without morphological changes indicative of muscle loss. This suggests that estradiol intervention effectively maintains the integrity of muscle structure and counteracts muscle loss caused by osteoporosis. The GSK group also exhibited well-formed muscle cells with an orderly arrangement and no signs of muscle loss, as observed morphologically. Similar to the estradiol group, the intervention in the GSK group significantly protected the structure and function of muscle tissue, demonstrating the potentially important role of this treatment in improving muscle quality (Fig. **4B**).

In the model group, the surface of the articular cartilage in the joint exhibited rough characteristics, with a small amount of chondrocyte shedding leading to the formation of fissures. Localized growth plates showed signs of deficiency, and a small number of chondrocytes and osteocytes exhibited necrosis and dissolution. Meanwhile, the formation of new cartilage was also observed in certain areas. In contrast, the control groups femoral joint cartilage surface was smooth, with no apparent abnormalities in chondrocytes. The trabecular bone and growth plate structures were intact, and no significant inflammatory cell infiltration was observed. In the GSK group, a small amount of chondrocyte necrosis and dissolution was visible in the local growth plate, but the overall structure of the articular cartilage and trabecular bone remained intact, with no obvious signs of inflammation. Comparatively, the estradiol groups articular cartilage also showed a rough surface, with local fissures accompanied by chondrocyte necrosis, dissolution, and a small amount of hemorrhage. Multiple deficiencies in the growth plate structure were observed in this group as well, but the trabecular bone structure remained intact, and no significant inflammatory cell infiltration was detected. These results indicate that the model group exhibited marked pathological changes in articular cartilage, whereas the intervention groups partly maintained the structural integrity of the bone tissue (Fig. **4C**).

#### 3.7.2 Immunohistochemical Staining of Muscle and Skeletal Tissues

**Fig. (5):**
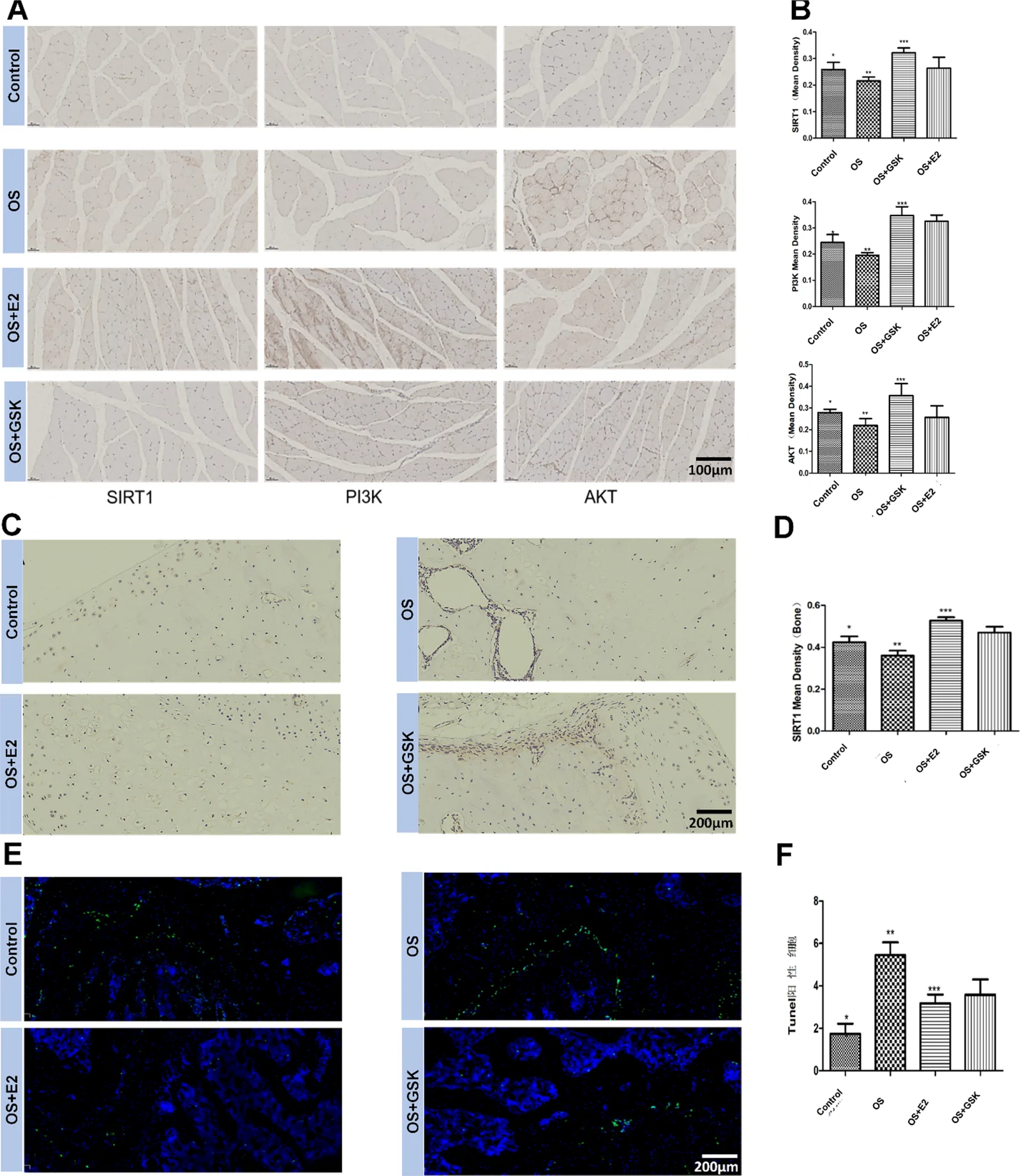
Effects of SIRT1, PI3K, and AKT Expression in Muscle and Bone Tissues, and Apoptosis Assessment in Cartilage. (**A**) Immunohistochemical analysis of SIRT1, PI3K, and AKT expression in muscle tissue sections. (**B**) Quantitative analysis of immunohistochemical staining in panel A, showing gene expression levels. The x-axis represents different group categories, while the y-axis indicates the relative gene expression levels of SIRT1, PI3K, and AKT. (**C**) Immunohistochemical expression of SIRT1 in bone tissue sections. (**D**) Quantitative analysis of immunohistochemical staining in panel C, displaying gene expression levels. The x-axis represents different group categories, while the y-axis shows the relative expression levels of SIRT1. (**E**) TUNEL staining to visualize apoptosis in cartilage cells within the femur. (**F**) Quantitative analysis of TUNEL staining in panel E, illustrating the number of positive apoptotic cells. The x-axis represents different group categories, while the y-axis shows the count of TUNEL-positive cells.

In the pectoralis muscle tissue of *rats* in the GSK intervention group, the expression levels of SIRT1, PI3K, and AKT proteins were significantly increased compared to the model group (*P* < 0.05), indicating that GSK intervention notably promoted the expression of these key regulatory factors. Immunohistochemical analysis showed that after GSK treatment, the PI3K/AKT signaling pathway was effectively activated, with a significant increase in the expression of related proteins (Fig. **5A** and **B**). This phenomenon reflects the critical role GSK plays in promoting muscle cell growth and survival. Compared to the model group, the expression of SIRT1 protein in the bones of *rats* in both the estradiol group and the GSK group was elevated (*P* < 0.05), suggesting that these two interventions similarly have positive effects on metabolism regulation and bone health promotion. Overall, these results suggest that GSK may exert a positive regulatory effect on improving muscle quality and overall health by activating the PI3K/AKT signaling pathway, while estradiol and GSK show promising potential in promoting bone health and maintaining muscle function (Figs. **5C** and **D**). These findings provide crucial experimental evidence for further exploration of the effects of GSK and other interventions on muscle and bone health.

#### 3.7.3 TUNEL Staining to Observe Apoptotic Cells

In the femurs of the control group, apoptotic cells were primarily located in the chondrocyte layer and hypertrophic layer, while a small number of scattered apoptotic osteocytes were also observed in the subchondral bone tissue. In contrast, the number of apoptotic cells in the model group increased significantly, appearing not only in the proliferative layer, chondrocyte layer, and hypertrophic chondrocyte layer, but also more prominently distributed among the subchondral osteocytes. This change indicates that the establishment of the osteoporosis model led to a more severe occurrence of cell apoptosis.

In the GSK and estradiol groups, apoptotic cells were still distributed in the chondrocyte layer and hypertrophic layer, but in relatively reduced numbers. Quantitative analysis based on TUNEL staining revealed that the number of apoptotic cells in cartilage and subchondral bone in the model group was significantly higher than that of the control group (*P* < 0.01). In contrast, the number of apoptotic cells in cartilage and subchondral osteocytes in the treatment groups was significantly lower than in the model group (*P* < 0.05) (Figs. **5E** and **F**). These results suggest that GSK or estradiol may effectively reduce apoptosis of chondrocytes in the proliferative layer, thereby decreasing the overall distribution of apoptotic cells. This finding provides essential experimental evidence for using GSK or estradiol as potential therapeutic approaches to alleviate osteoporosis-associated cell apoptosis.

### 3.8 GSK Regulates the Expression of Proteins Associated with Potential Pathways of Sarcopenia and Osteoporosis

To further investigate whether GSK regulates the PI3K/Akt/Bad signaling pathway, we conducted a detailed analysis of the expression of related proteins in mouse cartilage. The results showed that in the model group, both PI3K and AKT proteins were significantly activated and phosphorylated, indicating abnormal activation of this signaling pathway in the osteoporosis model. Additionally, phosphorylation of the BAD protein was also activated, promoting the formation of heterodimers that facilitate apoptotic processes, thereby exacerbating osteocyte apoptosis and functional impairment.

**Fig. (6):**
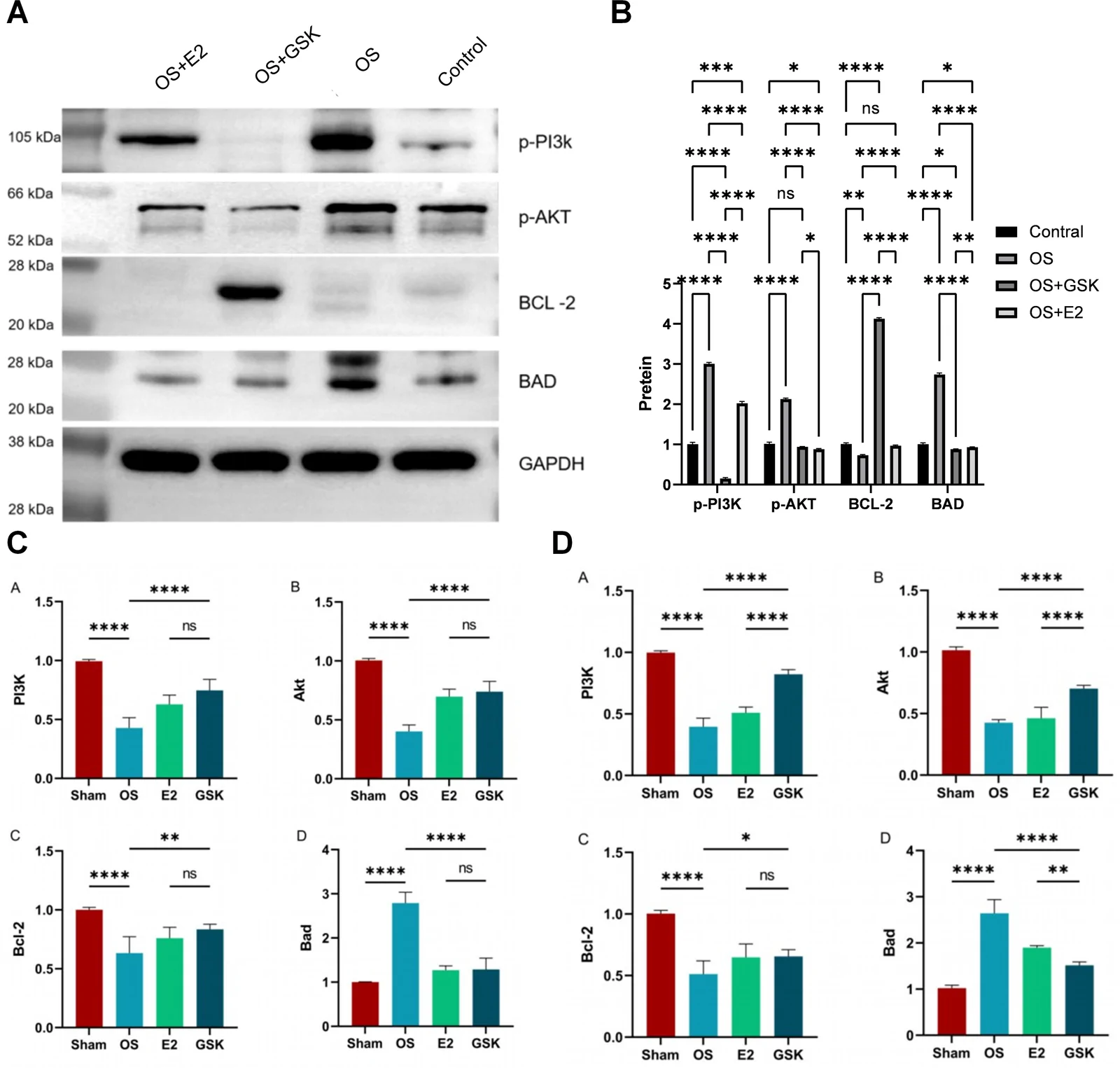
(**A** and **B**). GSK reduces chondrocyte apoptosis by modulating the PI3K/Akt/Bad signaling pathway: Western blot analysis showed that the phosphorylation levels of PI3K, AKT, and BAD were significantly increased in the model group, whereas GSK treatment inhibited these phosphorylation levels, concurrently increased the expression of the anti-apoptotic protein Bcl-2 and decreased the expression of the pro-apoptotic protein BAD, effectively preserving cartilage tissue structure and function. (The x-axis represents group categories, and the y-axis represents protein expression levels.) (**C** and **D**). GSK coordinately regulates apoptosis-related gene expression in bone (left) and muscle (right): RT-PCR analysis indicated that, compared with the model group, GSK treatment significantly upregulated the mRNA levels of PI3K, AKT, and Bcl-2 in both bone and muscle tissues while downregulating Bad mRNA expression, confirming that GSK exerts protective effects in both tissues by inhibiting PI3K/Akt/Bad pathway-mediated apoptosis (The x-axis represents grouping categories, and the y-axis represents gene expression levels).

In contrast, after GSK intervention, these adverse effects were significantly alleviated. Compared to the model group, GSK treatment markedly inhibited the phosphorylation levels of the PI3K/Akt signaling pathway, suggesting that GSK can effectively modulate the activity of this critical signaling cascade. Furthermore, we observed a significant increase in the expression of the anti-apoptotic protein Bcl-2, which enhanced cell survival capacity. Conversely, the expression of the apoptosis-related protein BAD was notably decreased, reflecting GSKs effective intervention in the apoptotic signaling pathway (Figs. **6A** and **B**).

Collectively, these results demonstrate that GSK suppresses the overactivation of the PI3K/Akt signaling pathway, reinforces anti-apoptotic mechanisms, reduces cell apoptosis, and thereby helps protect and maintain the structure and function of cartilage tissue (as shown in the Figure). This mechanism provides an important molecular basis for the application of GSK in the treatment of osteoporosis and related diseases.

### 3.9 GSK Regulates the Expression of Apoptosis-Related Genes in Bone and Muscle

We performed an in-depth analysis of mRNA levels of relevant pathways and apoptosis-related proteins in *rat* bone and muscle tissues using quantitative Reverse Transcription-Polymerase Chain Reaction (RT-PCR) technology. The results showed that, following Osteoporosis (OS) induction, the mRNA levels of PI3K, AKT, and the anti-apoptotic protein Bcl-2 were significantly decreased in the model group compared to the control group, reflecting a weakening of cell survival signals. Conversely, the expression level of the apoptosis-related protein Bad was significantly increased, indicating a significant increase in apoptosis.

However, compared to the model group, we observed a significant upregulation of PI3K, AKT, and Bcl-2 mRNA levels after GSK intervention, suggesting that GSK can effectively activate these protective signaling pathways. Simultaneously, GSK treatment also significantly downregulated the expression of the apoptotic protein Bad, further supporting the anti-apoptotic mechanisms (Figs. **6C** and **D**).

These results were highly consistent in both bone and muscle tissues, suggesting that GSK not only plays a role in preventing bone cell apoptosis but also effectively protects muscle cells. These findings indicate that GSK significantly inhibits PI3K/Akt/Bad signaling pathway-induced apoptosis of bone cells and muscle cells, providing an essential molecular basis and theoretical rationale for GSK's therapeutic potential in osteoporosis and related muscle atrophy diseases.

In this comprehensive study, we used an integrated multi-omics approach to investigate the molecular mechanisms by which Gushukang granules (GSK) intervene in sarcopenia and osteoporosis. Our findings elucidate the intricate relationships between key signaling pathways and GSKs therapeutic effects on muscle and bone health. Specifically, we confirmed that GSK regulates the PI3K/Akt signaling pathway, enhances protein synthesis, and reduces apoptosis, and it also effectively improves muscle mass and strength in osteoporotic animals. This work combines Traditional Chinese Medicine (TCM) practice with modern scientific methods, providing solid evidence for the treatment of sarcopenia osteoporosis. The contribution of this study is significant because it allows for a detailed molecular understanding of GSKs mechanisms of action and highlights its potential as a multifaceted therapeutic strategy for age-related musculoskeletal diseases. By integrating network pharmacology, metabolomics, and molecular biology techniques, we identified key active components and related targets, demonstrating GSKs ability to affect multiple pathways simultaneously. Rigorously validated animal experiments substantively bolster our results and enhance the feasibility of TCM formulations in modern medical practice.

Our study, while providing promising insights, acknowledges inherent limitations. The translational potential of our findings is constrained by the reliance on animal models, which, despite offering valuable mechanistic perspectives, cannot fully capture the intricate complexities of human musculoskeletal physiology. The multifaceted nature of GSK's therapeutic mechanisms necessitates comprehensive, large-scale human clinical trials to definitively validate our preliminary observations, integrating sophisticated analyses of genetic variability, environmental interactions, and personalized metabolic responses. Future research must adopt a holistic approach that bridges preclinical investigations with advanced translational strategies, ultimately developing nuanced, precision-medicine interventions for musculoskeletal health management.

Based on our findings and the limitations identified, we recommend that future research should, firstly, expand sample sizes and diversify study models by including more animal models and clinical cohort studies. Secondly, researchers should apply new technologies such as single-cell sequencing to probe cellular-level mechanisms in depth and combine multi-omics approaches to comprehensively reveal action pathways. Thirdly, human clinical trials should be conducted to assess safety and efficacy, as well as to explore individualized dosing regimens. Finally, the scope of application should be broadened to investigate the potential roles of Gushukang granules in other aging-related diseases and develop more precise TCM intervention strategies.

This study provides a model for the modernization of TCM and exemplifies the unique value of the TCM concept of “preventing disease before it occurs” in modern medical translation. By integrating traditional Chinese medical theory with modern biomedical research paradigms, we have not only provided new insights into treating muscle and bone atrophy but also opened new avenues for comprehensive interventions in chronic diseases of the elderly. Future research should deepen the understanding of its active components and mechanisms of action and explore combination therapies targeting multiple pathways in sarcopenia and osteoporosis. By promoting interdisciplinary collaboration and integrating traditional knowledge with modern scientific technologies, we can develop more effective and personalized interventions to address chronic diseases that severely affect the global aging population.

## 4 DISCUSSION

Despite our compelling findings, this study has notable limitations. While animal models offer valuable insights into the biological mechanisms of GSK, their inherent differences from human physiology limit the direct applicability of our results. Furthermore, although we identified numerous pathways and components affected by GSK, further research involving larger human cohorts and well-designed clinical trials is essential to thoroughly elucidate its mechanisms and validate its therapeutic benefits. Additionally, the multifactorial nature of musculoskeletal decline underscores the need to explore other influencing factors, such as genetic predisposition and environmental variables, to develop more comprehensive and effective treatment strategies [39, 40].

To advance our understanding and translational potential, we propose a strategic, multi-dimensional research framework. Future investigations should transcend conventional methodological boundaries by integrating cutting-edge technologies, such as single-cell sequencing, spatial transcriptomics, and comprehensive multi-omics approaches, to dissect the intricate molecular mechanisms underlying Gushukang granules' therapeutic effects. Concurrently, we advocate for rigorous, large-scale clinical trials that not only validate safety and efficacy but also explore personalized intervention strategies, considering individual genetic architectures, metabolic variabilities, and age-related physiological diversity. Moreover, our research trajectory should extend beyond musculoskeletal applications, systematically investigating the granules' potential therapeutic implications across broader aging-related pathological spectrums, thereby repositioning traditional Chinese medicine within a precision medicine paradigm that bridges historical wisdom with contemporary scientific methodologies.

This study exemplifies a pioneering approach to the modernization of Traditional Chinese Medicine (TCM), highlighting the inherent value of the TCM philosophy of “preventing disease before it occurs” in the context of contemporary medical translation. By harmonizing traditional Chinese medical concepts with advanced biomedical research frameworks, we introduce innovative strategies for addressing muscle and bone atrophy. Future investigations should prioritize a comprehensive understanding of the active components in Gushukang granules and elucidate their underlying mechanisms of action. Additionally, exploring combination therapies that target multiple pathways involved in sarcopenia and osteoporosis could significantly enhance therapeutic efficacy. Fostering interdisciplinary collaboration that blends traditional wisdom with cutting-edge scientific technologies will not only facilitate the development of more effective, personalized interventions for chronic diseases but also address the pressing health challenges faced by the aging global population holistically.

## CONCLUSION

In this comprehensive study, we utilized an integrated multi-omics approach to investigate the molecular mechanisms by which Gushukang granules (GSK) treat sarcopenia and osteoporosis. Our findings elucidate the intricate relationships between key signaling pathways and GSKs therapeutic effects on muscle and bone health. Specifically, we confirmed that GSK regulates the PI3K/Akt signaling pathway, enhances protein synthesis, and reduces apoptosis. This work integrates Traditional Chinese Medicine (TCM) with modern scientific methods, providing substantial evidence for the efficacy of GSK in treating sarcopenia osteoporosis.

The significance of this study lies in its detailed molecular understanding of GSKs mechanisms of action, highlighting its potential as a multifaceted therapeutic strategy for age-related musculoskeletal diseases. Our investigation also demonstrates GSKs ability to affect multiple pathways simultaneously. The rigorously validated animal experiments support our results and enhance the applicability of TCM formulations in contemporary medical practice.

## Figures and Tables

**Table 1:** UPLC-MS/MS information of GSK major compounds.

Core Target	PBD ID	Binding Energy(kcal/mol)		
-	-	quercetin	luteolin	daidzein
INS	7S4Y	-5.8	-6.3	-6.5
TP53	7BWN	-8.5	-8.6	-8.3
TNF	1A8M	-5.7	-6.9	-6.1
AKT1	7NH5	-7.9	-10.1	-7.5
IL6	1IL6	-7.3	-6.3	-6.7

**Table 2:** Chemical composition analysis results of GSK Granule samples by ultra-performance liquid chromatography-tandem mass spectrometry (UPLC-MS/MS).

**Compound**	**Rt (min)**	**m/z Actual Value**	**m/z Theoretical Value**	**ppm**	**Adduct Ion**	**Structural Type**
Glycyrrhizin Acid	20.08875	469.3317	469.3323	-1.27841354	[M-H]-	Triterpene
Linoleic Acid	23.25238	277.2182	277.2173	3.24654009	[M-H]-	Polyunsaturated Fatty Acid
Elderberry Oil Acid	24.25693	279.2322	279.233	-2.864999094	[M-H]-	Long-chain Fatty Acid
Ursolic Acid	23.82358	455.3519	455.3531	-2.63532446	[M-H]-	Triterpene
Corosolic Acid	20.7892	473.3564	473.3625	-12.88669594	[M+H]+	Triterpene
Oleanolic Acid	15.04327	455.3471	455.352	-10.76102165	[M+H]+	Triterpene
Ricinoleic Acid	20.92408	297.2437	297.2435	0.672848575	[M-H]-	Hydroxy Fatty Acid
Jasmine Acid	6.649117	211.1339	211.1329	4.736330831	[M+H]+	-
Madecassoside Acid	17.40837	487.3427	487.3429	-0.41038883	[M-H]-	Triterpene
Ivy Saponin Aglycone	20.88015	471.3471	471.348	-1.909420892	[M-H]-	Triterpene

**Table 3:** Comparison of body weight and gastrocnemius muscle among groups of *rats* (Mean ± SD).

**Group**	**Body Weight (g)**	**Gastrocnemius Muscle (g)**	**Gastrocnemius Muscle/Body Weight (%)**
Model Group	320 ± 18.6*	0.72 ± 0.05*	0.23 ± 0.02*
Control Group	486 ± 23.4	1.49 ± 0.11	0.30 ± 0.06
Bone Health Granule Group	453 ± 19.6**	1.32 ± 0.09**	0.29 ± 0.05**
Estradiol Group	436 ± 24.8***	1.18 ± 0.06***	0.27 ± 0.02***

## Data Availability

All data generated or analyzed during this study are included in this published article.

## LIST OF ABBREVIATIONS

GSK: Gushukang Granules
OP: Osteoporosis
SP: Sarcopenia
OS: Osteosarcopenia
TCM: Traditional Chinese Medicine
PBS: Phosphate-Buffered Saline
PPI: Protein-Protein Interaction
GO: Gene Ontology
KEGG: Kyoto Encyclopedia of Genes and Genomes
DL: Drug-likeness
OB: Oral Bioavailability
UPLC-MS/MS: Ultra-Performance Liquid Chromatography-Tandem Mass Spectrometry
qRT-PCR: Quantitative Reverse Transcription-Polymerase Chain Reaction
ELISA: Enzyme-Linked Immunosorbent Assay
IL: Interleukin
GM: Glycidyl Methacrylate
EPO: Erythropoietin
DNA: Deoxyribonucleic Acid
PI3K: Phosphatidylinositol 3-Kinase
Akt: Protein Kinase B
BCG: Bacillus Calmette Guérin
SRF: Serum Response Factor
VEGF: Vascular Endothelial Growth Factor
TGF: Transforming Growth Factor
BMP: Bone Morphogenetic Proteins
MAPK: Mitogen-Activated Protein Kinase
PIP: Phosphatidylinositol
